# The Liver Pre-Metastatic Niche in Pancreatic Cancer: A Potential Opportunity for Intervention

**DOI:** 10.3390/cancers14123028

**Published:** 2022-06-20

**Authors:** Peter Gumberger, Bergthor Bjornsson, Per Sandström, Linda Bojmar, Constantinos P. Zambirinis

**Affiliations:** 1 Department of Surgery, Linköping University, 58183 Linköping, Sweden; peter.gumberger@regionostergotland.se (P.G.); bergthor.bjornsson@liu.se (B.B.); per.sandstrom@liu.se (P.S.); 2 Department of Biomedical and Clinical Sciences, Linköping University, 58183 Linköping, Sweden; linda.bojmar@liu.se; 3 Children’s Cancer and Blood Foundation Laboratories, Departments of Pediatrics, and Cell and Developmental Biology, Drukier Institute for Children’s Health, Meyer Cancer Center, Weill Cornell Medicine, New York, NY 10021, USA; 4 Division of Surgical Oncology, Rutgers Cancer Institute of New Jersey, New Brunswick, NJ 08901, USA

**Keywords:** pancreatic cancer, PDAC, pre-metastatic niche, liver metastasis, immunotherapy

## Abstract

**Simple Summary:**

Patients with pancreatic cancer have a very poor chance of long-term survival. This is usually due to advanced disease at the time of diagnosis, which commonly includes occult or clinically obvious liver metastases. Emerging evidence suggests that organs that develop metastases exhibit microscopic changes that favor metastatic growth, collectively known as “pre-metastatic niches”. Such pre-metastatic niches result from various signals originating from the primary pancreatic tumor that reprogram immune and other cells in the liver and other organs, thus enabling the growth of cancer cells once they spread. In this review, we summarize the latest discoveries regarding the liver pre-metastatic niche in pancreatic cancer. We are optimistic that intensified future research will help to reveal powerful diagnostic markers and targetable therapeutic pathways, which will ultimately benefit patients.

**Abstract:**

Cancer-related mortality is primarily a consequence of metastatic dissemination and associated complications. Pancreatic ductal adenocarcinoma (PDAC) is one of the most lethal malignancies and tends to metastasize early, especially in the liver. Emerging evidence suggests that organs that develop metastases exhibit microscopic changes that favor metastatic growth, collectively known as “pre-metastatic niches”. By definition, a pre-metastatic niche is chronologically established before overt metastatic outgrowth, and its generation involves the release of tumor-derived secreted factors that modulate cells intrinsic to the recipient organ, as well as recruitment of additional cells from tertiary sites, such as bone marrow—all orchestrated by the primary tumor. The pre-metastatic niche is characterized by tumor-promoting inflammation with tumor-supportive and immune-suppressive features, remodeling of the extracellular matrix, angiogenic modulation and metabolic alterations that support growth of disseminated tumor cells. In this paper, we review the current state of knowledge of the hepatic pre-metastatic niche in PDAC and attempt to create a framework to guide future diagnostic and therapeutic studies.

## 1. Introduction

Efficient treatment of pancreatic ductal adenocarcinoma (PDAC) remains a challenge. A 5-year survival rate of 9% ranks it seventh globally in terms of cancer-related deaths [1,2]. It is expected that the annual incidence will increase from 458,918 cases in 2018 to around 814,235 cases in 2040 [2]. It is also projected that within the next two decades, PDAC will rank second and third in terms of cancer-related deaths in the US and the EU, respectively [2,3]. Eligibility for surgery, which currently offers the only real possibility for long-term survival or cure, often depends on the presence of metastasis.

More than 50% of PDAC patients have distant metastasis at the time of diagnosis [1], and the majority of those metastases occur in the liver and lymph nodes [4]. Stephen Paget’s “seed and soil” theory suggests that predisposition—rather than sheer coincidence—is a prime determinant of metastatic outcome. In particular, distinct cancer cells equipped with specific phenotypic features possess metastatic potential, whereas secondary organs feature specific characteristics in their microenvironment that facilitate colonization by disseminated tumor cells (DTCs) and enable their growth into metastases [5].

Initially proposed in 2005, the term “pre-metastatic niche” implies the presence of an amenable microenvironment induced by a primary tumor in a secondary organ that features favorable conditions for and increases the probability of metastasis [6]. Current understanding proposes a complex interplay in the preparation of the pre-metastatic niche between tumor-derived factors, tumor-mobilized bone-marrow-derived cells and secondary-organ-intrinsic components [6]—in other words, a framework through which the primary tumor itself remotely prepares the “soil”.

An optimal experimental setup for the study of the pre-metastatic niche requires an established source of tumor-derived secreted factors (TDSFs), the absence of tumor cells in the soon-to-be metastatic organ, immunocompetence and traceable cancer cells that home into the pre-metastatic niche, concomitantly providing enough time for interrogation of the mechanisms involved in its formation [7]. There is a scarcity of studies conducted in accordance with the aforementioned experimental conditions.

In this review, we focus on the liver during pancreatic carcinogenesis and metastagenesis. We summarize the latest discoveries with respect to the pre-metastatic niche and attempt to incorporate them in specific mechanistic categories. With such a framework in mind, we discuss areas worthy of additional elucidation that may lead to improved understanding of the pre-metastatic niche concept and contribute to the identification of diagnostic and therapeutic opportunities.

## 2. Drivers of the Pre-Metastatic Niche

During carcinogenesis, cancer cells within the primary tumor modulate the surrounding tissue to shape the tumor microenvironment (TME). In parallel, distant organs are also influenced by the primary tumor, resulting in the establishment of pre-metastatic niches and subsequent metastases. Such changes in the microenvironment are achieved through a multitude of cellular and molecular components that engage in different pathways to promote metastasis. In particular, TDSFs such as extracellular vesicles (EVs), soluble growth factors, chemokines, cytokines and circulating cells that home into the pre-metastatic organ, such as tumor-mobilized bone-marrow-derived cells (BMDC), all contribute to pre-metastatic niche formation. These phenomena likely stem both from stochastic events within the neoplastic cells, which alter their behavior to their advantage in the context of natural selection, as well as from deranged homeostatic responses within the TME [8].

The introduction of “the hallmarks of cancer” has served as an important foundation for the organization and conceptualization of the most important aspects of carcinogenesis and disease evolution [9]; however, because the concept of the pre-metastatic niche is relatively novel, a broadly accepted framework for its aspects is remains lacking. A recent review suggested six potential hallmarks of the pre-metastatic niche: reprogramming, inflammation, immunosuppression, organotropism, lymphangiogenesis and angiogenesis/vascular permeability [10]. One caveat is that different cancer types have different metastatic propensities, modes of dissemination and, potentially, very diverse underlying biologic events, making the creation of a unifying framework more challenging. Moreover, several of the putative mediators of pre-metastatic niche formation may exert pleiotropic effects that correspond to more than one hallmark (Table 1); therefore, assigning them to one category may lead to inadvertent neglect of additional modes of action. In subsequent paragraphs, we group factors into hallmark categories to facilitate their description; however, we caution the reader to keep an open mind, given this overlap.

### 2.1. Reprogramming

#### 2.1.1. Stromal Reprogramming

Remodeling of the TME and secondary organ stroma plays a crucial role in development of metastasis. Although previously, the stroma was regarded as a mere mass of connective tissue that supports organ architecture, this perception has more recently shifted to encompass a structure with a wide array of properties that range from involvement in matrix–cell adhesion to cell–cell signaling and cell behavior control by altering mechanical and biochemical properties of the stroma [24].

##### Tumor Microenvironment Co-Determines Pre-Metastatic Niche Formation

PDAC exhibits intense desmoplastic behavior characterized by a robust elaboration of the extracellular matrix (ECM). The immediate TME is characterized by increased numbers of activated pancreatic stellate cells (PSCs) and cancer-associated fibroblasts (CAFs), as well as several other cell types, including endothelial cells, pericytes and neurons, in addition to proteins, including collagen I, III and IV; fibronectin (FN); and hyaluronic acid [25]. Interestingly, recent studies have subclassified CAFs into pro-tumoral inflammatory CAFs (iCAFs) and anti-tumoral myofibroblastic CAFs (myCAFs) based on differences in their cytokine and marker expression profiles, respectively [26,27]. A third subgroup called mesenchymal stem cell CAFs (mscCAFs) led to increased liver metastasis through granulocyte-macrophage colony-stimulating factor (GM-CSF) signaling when co-injected with human PDAC cells in an orthotopic mouse model [28]. Another study identified a subset called antigen-presenting CAFs (apCAFs), which have a potential immunosuppressive role in PDAC [29]. Therefore, CAFs not only support cancer cell growth and egress from the primary tumor but may also escort cancer cells to distant organs to enable the establishment of micrometastases; in other words, they may behave as DTCs themselves.

Tumor-associated macrophages (TAMs), myeloid-derived suppressor cells (MDSCs) and regulatory T cells (Treg) contribute to an immunosuppressive milieu within the TME that impedes CD8^+^ T-cell function, thus promoting tumor growth, invasion, migration and metastasis [30,31]. The stroma surrounding the tumor is thought to mainly support primary tumor growth; nevertheless, there are indications that components of the TME are involved in the release of pre-metastatic-niche-promoting factors, including cytokines, fibroblasts and macrophages [32].

**Figure 1:**
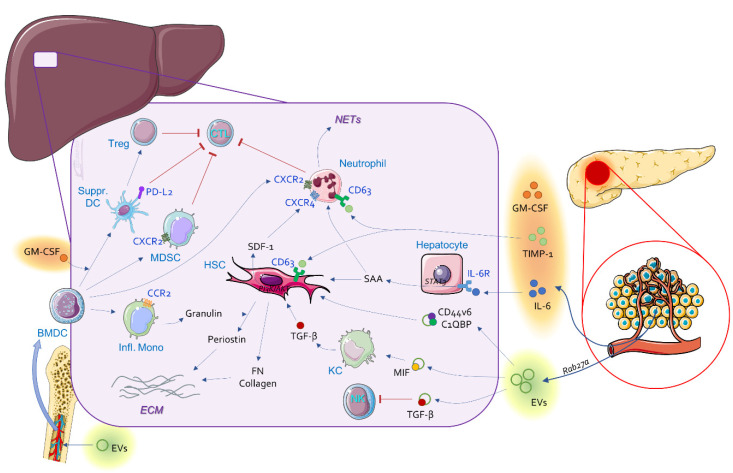
Mechanisms of PDAC pre-metastatic niche formation in the liver. BMDC, bone-marrow-derived cells; CTL, cytotoxic T lymphocytes; ECM, extracellular matrix; EVs, extracellular vesicles; FN, fibronectin; GM-CSF, granulocyte-macrophage colony-stimulating factor; HSC, hepatic stellate cells; Infl. Mono, inflammatory monocytes; KC, Kupffer cells; MDSC, myeloid-derived suppressor cells; MIF, macrophage migration inhibitory factor; NK, natural killer cells; SAA, serum amyloid A; SDF-1, stromal-cell-derived factor 1 (CXCL12); Suppr. DC, suppressive dendritic cells; TGF-β, transforming growth factor-β; TIMP-1, tissue inhibitor of metalloproteinases; Treg, regulatory T cells.

The liver microenvironment is composed of two main types of parenchymal cells—hepatocytes and biliary epithelial cells—as well as a variety of non-parenchymal cells, including liver sinusoidal endothelial cells (LSECs), hepatic stellate cells (HSCs) and resident immune cells, such as dendritic cells (DCs), resident macrophages known as Kupffer cells (KCs), natural killer (NK) cells, NKT cells, B cells and T cells. Emerging evidence suggests that modulation of the phenotype of these cells combined with recruitment of BMDCs, such as inflammatory monocyte-derived macrophages, shapes the hepatic pre-metastatic niche and precedes the occurrence of clinically detectable metastasis. There are signs that this process occurs in a similar fashion to TME remodeling. Ultimately, a pre-metastatic niche dominated by activated HSCs, metastasis-associated fibroblasts (MAFs), metastasis-associated macrophages (MAMs) and many other facilitators of metastases represents an amicable microenvironment for incoming tumor cells [33] (Figure 1).

#### 2.1.2. Soluble Factors

The first study to point out the seminal role of TDSFs in pre-metastatic niche formation showed that mice that intravenously inoculated with Lewis lung carcinoma (LLC) or B16 melanoma cells released tumor-derived vascular endothelial growth factor (VEGF) and placental growth factor (PLGF)—molecules involved in angiogenesis—which in turn stimulated BMDCs, specifically VEGFR1^+^ hematopoietic progenitor cells (HPCs) [11]. Mobilized BMDCs established clusters in future metastatic sites of the lungs and liver. Those niches were characterized by upregulation of FN expression, production of matrix metalloproteinase 9 (MMP-9) and upregulation of the chemokine stromal-cell-derived factor 1 (SDF-1/CXCL12). BMDC expression of transmembrane receptor VLA-4 (also known as integrin α4β1) and inhibitor of differentiation 3 (Id3, a protein involved in regulation of the immune response in melanomas [34]) enhanced pre-metastatic cluster formation by augmenting adhesion among HPCs and migration of HPCs.

Matrix metalloproteinases (MMPs) have been widely described to drive tumor progression and metastasis through decomposition of the ECM, thus enabling stromal remodeling and egress of cancer cells from the primary tumor into the blood stream. Moreover, they may facilitate release of ECM-bound pro-tumorigenic factors, such as TGF-β [35]. MMPs may originate both from cancer cells and immune cells or other stromal cells (e.g., PSCs and HSCs), adding an extra layer of complexity to the investigation of their role. For example, myeloid cells recruited within the PDAC microenvironment by KRAS-mutant pancreatic epithelial cells secrete IL-6, which leads to secretion of MMP-7 by epithelial cells in a STAT3-dependent manner [36,37].

Specific MMPs may play conflicting roles in carcinogenesis and metastasis. MMP-9 was described as a two-faced coin that, depending on the context, may have pro-tumorigenic or pro-metastatic attributes or engage tumor-inhibitory pathways [38]. In MMP-9 knockout mice, MMP-9 was found to have a pro-tumorigenic and pro-metastatic role [39]. Specifically, MMP-9-deficient mice had significantly increased IL-6 production by bone marrow cells, which seemed to be a compensatory mechanism for MMP-9 deficiency. This in turn led to activation of the STAT3 pathway, leading to increased migration, proliferation and invasion of KPC-derived PDAC cells in vitro. Blockage of the IL-6 receptor (IL-6R) abolished these effects. Interestingly, MMP-9 ablation in KPC-derived PDAC cells in vitro led to decreased migration, invasion and proliferation. On the contrary, increased levels of systemic TIMP-1, an endogenous inhibitor of MMPs, led to pre-metastatic niche initiation in the liver [13].

The aforementioned studies showcase how the clinical application of systemically acting inhibitors may be complicated by the multifunctional nature of MMPs and TIMPs, which may lead to opposing effects depending on cellular type, organ or use of co-inhibitors. These conflicting and context-dependent functions of MMPs may justify the negative results of randomized trials testing novel MMP inhibitors in advanced PDAC [40,41]. Regardless, similar to what is observed within primary tumors, the hepatic microenvironment is also remodeled by MMPs, as has been described in the context of liver inflammation and fibrosis [42]. Notably, recruited monocytes that acquire a housekeeping role secrete MMP-9, which can dampen activated inflammatory pathways and limit fibrosis [43]. Such pathways may be hijacked by TDSFs and lead to immunosuppression within the liver, which in turn enables metastasis.

Both IL-6 and STAT3 act as core mediators of inflammatory response and can promote PDAC progression and metastasis [44]. Among their multiple effects is the gene activation and subsequent production of serum amyloid A (SAA), an acute phase protein produced in the liver [45]. In the KPC mouse model of spontaneous PDAC [46], STAT3 was found to be activated in 80–90% of hepatocytes compared to only 2% in control mice. This in turn led to upregulation of genes encoding myeloid chemoattractants, such as SAA. IL-6 was shown to be expressed by non-malignant α-SMA^+^ stromal cells within the primary tumor, likely acting on hepatocytes through the IL-6/JAK1/STAT3 axis. IL-6-deficient mice exhibited significantly decreased hepatocyte STAT3 activity and reduced SAA expression, resulting in fewer metastases. In particular, certain features of the pre-metastatic niche, including hepatic parenchymal FN and collagen I production, as well as Ly6G^+^ myeloid cell infiltration, were curtailed in SAA-deficient mice, indicating a crucial role of SAA in hepatic pre-metastatic niche formation [12].

Building on previous study results [13], a more recent study showed that TIMP-1 levels are increased both in the blood and pancreas in mouse models with premalignant pancreatic lesions, such as chronic pancreatitis, pancreatic intraepithelial neoplasia (PanIN) and PDAC. TIMP-1 is a PI3K-dependant activator of HSCs. Inhibition of TIMP-1; its receptor, CD63; or PI3K suppressed HSC/SDF-1/CXCR4-mediated neutrophil accumulation in the liver, which in turn led to significantly decreased hepatic homing and outgrowth of PDAC cells in the liver [14]. This suggests that pre-metastatic niche preparation begins during pre-malignant pancreatic events and that prevention of neutrophil accumulation may inhibit this process, thus reducing the metastatic burden.

#### 2.1.3. Extracellular Vesicles

Exosomes are extracellular vesicles with a lipid bilayer membrane ranging from 40 to 150 nm in diameter and originating from the endosomal compartment. They carry proteins, lipids and nucleic acids and can contribute to intercellular communication in the normal state, as well as in various disease states. Exosomes have been shown to be secreted by multiple cell types within the tumor, including cancer cells and stromal cells, and act as drivers of tumorigenesis and metastatic progression [47,48].

In a preclinical study, exosomes isolated from the murine PDAC cell line PAN02 have been implicated in the formation of the hepatic pre-metastatic niche when injected intravenously. Through a cascade that involves their uptake by hepatic macrophages (KCs), they promote the activation of pathways involved in ECM remodeling. Specifically, the chemokine macrophage migration inhibitory factor (MIF) contained in tumor-derived exosomes led to the release of TGF-β by KCs, which in turn acted on HSCs, resulting in the production of FN and retention of additional BMDCs in the liver. These alterations in the hepatic microenvironment were sufficient to augment metastatic colonization by PAN02 cells, consistent with pre-metastatic niche formation. The pro-metastatic effects of PAN02-derived exosomes were reversed by depletion of macrophages or the use of FN knockout models [16].

The above-noted findings were supported by another study investigating exosomal influence on hepatic pre-metastatic niche formation. “Education” of mice with exosomes derived from PAN02 cells with high versus low metastatic potential led to an increased accumulation of CD11b^+^ and CD45^+^ HPCs in the hepatic pre-metastatic sites in the former compared to the latter and a control group, consistent with the high metastatic behavior of the parental cell line [17]. Furthermore, FN, S100A8 and S100A9 production were upregulated in the livers of exosome-educated C57BL/6 mice, and the frequency of MDSCs in the peripheral blood increased.

In a more recent study, red fluorescent protein (RFP)-labelled human Mia-PaCa-2 PDAC cells were injected intrasplenically into nude mice [49]. Beforehand, those cells were transduced with pCT-CD63-green fluorescent protein (GFP), an exosome marker, to allow for later visualization of exosomes by color-coded imaging. GFP-transduced exosomes were then observed in liver-resident KCs present within liver metastases. Interestingly, the same exosomes were observed in both lungs and bone marrow of nude mice, although without clinically detectable metastases. This points to exosome-independent factors in different organic milieus that seem to play a crucial role in the pro-metastatic potential of exosomes. A serious limitation of this study is the use of nude mice, which are significantly immunocompromised and therefore cannot recapitulate the full contribution of the immune system in pre-metastatic niche formation.

CD44v6, an adhesion molecule and marker for cancer-initiating cells in PDAC, has previously been described as an exosome-co-dependent promoter of the pre-metastatic niche [50,51]. CD44v6 was identified in a complex with C1q binding protein (C1QBP) within PAN02- and KPC-derived exosomes, which were taken up by desmin^+^ HSCs and KCs. Exosome fusion with HSCs led to upregulation of insulin-like growth factor-1 (IGF-1) and its downstream PI3K/AKT pathway. Blockage of the IGF-1 receptor reversed previously observed HSC activation, as well as the associated increase in FN and collagen I production. Inhibition of CD44v6 and C1QBP, either as a complex or separately, impeded exosome-induced liver fibrosis and liver metastasis in a mouse model. Furthermore, high exosomal CD44v6/C1QBP expression in human tissue and blood samples was shown to predict liver metastasis and poor survival in PDAC patients [52].

Rab27a is a GTPase known to play a pro-tumorigenic and pro-metastatic role in multiple cancer types through involvement in maturation, secretion and trafficking of exosomes, as well as MMP9 [53]. Knockdown of Rab27a in orthotopically implanted KPC cancer cells (shRab27a-KPC) altered the liver microenvironment by decreasing MDSC frequencies, including CD11b^+^Ly6C^+^Ly6G^+^ granulocytic cells, CD11b^+^Ly6C^+^Ly6G^−^ monocytic cells and CD11b^+^Ly6C^−^Ly6G^−^ macrophages compared to the liver microenvironment in mice inoculated with control KPC cells (scr-KPC) [21]. This phenomenon can possibly be explained by decreased MDSC mobilization resulting from diminished exosome stimulation, although the exact pathways remain unclear. Furthermore, intravenously injected extracellular vesicles (EV) from supernatant of scr-KPC cells increased the intrahepatic CD11b^+^ F4/80^+^ macrophage population but only slightly increased the intrahepatic CD11b^+^ Gr1^+^ neutrophil population in wild-type control mice compared to scr-KPC mice. Repletion of EVs in shRab27a-KPC mice partially restored MDSC levels but not to the levels encountered in scr-KPC mice, indicating that non-EV-mediated mechanisms play a role in hepatic MDSC expansion. Intriguingly, Rab27a-deficient tumors were more locally invasive compared to their scr counterparts, as suggested by their irregular borders.

In summary, tumor-derived exosomes appear to play a crucial role in pre-metastatic niche formation. They are primarily taken up by KCs, as well as HSCs, thereby activating an array of pathways that ultimately lead to ECM remodeling and reprogramming of the immune microenvironment of the liver. Additional cells that may have a role in exosome-mediated pre-metastatic niche formation but have yet to be studied include the liver sinusoidal endothelial cells (LSECs), which are highly phagocytic and have antigen-presenting cell and immunomodulatory capacities [54,55].

#### 2.1.4. Metabolic Reprogramming

The hypovascular, hypoxic and highly fibrotic microenvironment of PDAC imposes selection pressure, which promotes modification of the cancer cell metabolism in order to meet the increased energy requirements, as well as the scarcity of certain metabolites essential for tumor growth. In contrast to normal cells, which, by means of the tricarboxylic acid cycle and oxidative phosphorylation, produce energy, cancer cells often make use of the “Warburg effect”, which implies a preferential shift of the glycolytic pathway towards the anaerobic route, leading to lactate production, even under normoxic conditions. This “facultative aerobic glycolysis” fuels rapid and aggressive proliferation [56].

Mutant KRAS in pancreatic cancer cells was shown to downregulate expression of hormone-sensitive lipase (HSL), an enzyme involved in mobilization of stored triglycerides [57]. This in turn leads to storage of lipids in lipid droplets, which are later catabolized by lipolysis during tumor invasion and metastasis formation. C57BL/6 mice orthotopically injected with HSL-overexpressing KPC cells showed reduced tumor burden and liver metastasis compared to control mice bearing KPC tumors with wild-type HSL. In support of these findings, primary tumor and metastatic tissue from human pancreatic cancer patients were found to exhibit decreased HSL expression compared to normal adjacent tissue. This pro-metastatic process is relevant, as it demonstrates another possible window of opportunity for intervention in metastatic progression. Additionally, the question arises as to whether KRAS exerts metabolic influence over pre-metastatic niche cells in preparation for future metastatic growth.

Another study showed that co-culture of H6c7 human immortalized pancreatic ductal epithelial cells (PDECs) with activated HSCs but not quiescent HSCs were characterized by reduced expression of succinate dehydrogenase B (SDHB), which was associated with an increase in cancer stem cell properties of PDEC, including a metabolic change from oxidative phosphorylation to glycolysis. This suggests a tumor-suppressive role of SDHB, although the exact mechanisms leading to this phenotypical shift of PDEC when exposed to activated HSCs remain unclear [58]. More importantly, it demonstrates how the pre-metastatic niche may support pro-metastatic metabolic processes in DTCs (even in the pre-malignant stage).

### 2.2. Inflammation

Inflammation within the primary tumor microenvironment can set off signaling pathways that support pre-metastatic niche formation. Some of these pathways are shared with the pathways mentioned in the “Stromal remodeling section”. From the authors’ perspective, such crosstalk of the primary tumor with the pre-metastatic niche may occur through either (i) TDSF-mediated activation of tissue-resident immune cells already present at future metastatic sites; (ii) TDSF-mediated activation and mobilization of BMDCs, which in turn reach distant organs to prime the pre-metastatic niche [6,59]; or (iii) migration of immune cells (or potentially other non-cancerous cells) from the primary tumor to future metastatic sites, either before the arrival of DTCs or at the same time.

#### 2.2.1. Proinflammatory Mediators Recruit Myeloid Cells with and Invoke Pro-Metastatic Changes in the Liver

CXCR2, a member of the chemokine receptor family expressed by neutrophils and MDSCs, is known to play a role in their recruitment to sites of inflammation, as well as within tumors [60,61]. Human PDAC tumors overexpress CXCR2, and its activation by certain CXC chemokines has tumor- and inflammation-promoting consequences. CXCR2 inhibition with either pepducin, a short peptide that interferes with CXCR2 signaling, or CXCR2 small-molecule inhibitor (CXCR2 SM) halted formation of liver metastasis in KPC mice. Interestingly, the same results were recapitulated by both CXCR2 deletion and depletion of Ly6G^+^ cells using the murine anti-Ly6G antibody 1A8 [18]. Furthermore, concurrent CXCR2 inhibition and gemcitabine treatment led to decreased numbers of bone-marrow-derived F4/80^+^ macrophages and NIMP1^+^ neutrophils, as well as S100A9^+^ cells, in the livers of KPC mice compared to untreated control KPC mice. These findings imply that CXCR2 inhibition may be an attractive strategy for interruption of pre-metastatic myeloid cell migration to the liver, it may contribute to pre-metastatic niche formation. Furthermore, there may be an opportunity for synergy with existing immunotherapies, such as immune checkpoint inhibitors (ICI), as combined CXCR2 and PD-1 inhibition led to an overall increase in CD4^+^ helper T cells and CD8^+^ cytotoxic T cells, as well as CD3^+^ T cells.

Activated HSCs have been shown to assume a pro-metastatic phenotype, in part through engagement of inflammatory pathways [62]. We previously mentioned studies that implicate TDSFs, including TIMP-1 and TGF-β, as promoters of HSC activation in the pre-metastatic niche setting. In syngeneic mouse models of PDAC, activated HSCs were shown to promote proliferation of previously dormant pre-neoplastic H6c7-Kras human PDEC in the liver, whereas quiescent HSCs promoted a quiescent-associated phenotype (QAP) of PDEC. Younger mice presented with higher IL-8, which seemed to contribute to the QAP in PDECs, whereas older mice had higher numbers of activated HSCs and increased expression of VEGF, which supported reversion of quiescent PDEC status. These observations implicate age-related inflammatory changes in the hepatic microenvironment as contributors to metastatic outgrowth, which could be reversed by inhibition of VEGF [63]. This study seems to deliver a partial answer as to how increased TIMP-1 levels observed in conditions such as chronic pancreatitis and PanIN may reinforce inflammatory changes within the liver, which later contribute to metastatic outgrowth.

A previous study showed that pancreatic cancer produces CCL2, which leads to infiltration of CCR2^+^ macrophages and disease progression. In mice, subcutaneous injection of a CCR2 inhibitor (PF-04136309) ablated inflammatory monocytes and macrophages not only in the primary tumor but also in the pre-metastatic niche, leading to decreased tumor growth and metastases, as well as improved anti-tumor immunity [64].

Emphasizing the importance of macrophages as key players in the hepatic metastatic process, inflammatory monocyte-derived CD11b^+^ F4/80^+^ Ly6G^−^ CCR2^+^ MAMs, which are recruited from the BM, were shown to induce activation of HSCs via granulin secretion [19]. Stimulation by granulin leads to liver fibrosis and metastatic growth in wild-type mice inoculated with KPC cells as opposed to KPC inoculation of granulin-deficient mice. Subsequent extracellular matrix remodeling occurred by HSC protein secretion, including periostin. Considering the overarching model of pre-metastatic niche formation, this study did not model a clear pre-metastatic window, nor did it determine specific TDSFs initiating MAM recruitment to the liver; however, it demonstrates the crosstalk between inflammatory pathways and pre-metastatic organ stromal reprogramming.

In a recent study, bioengineered scaffolds were used to mimic the pre-metastatic niche and were implanted subcutaneously, followed by orthotopic inoculation with KPC-derived PDAC cells. The control group received subcutaneous scaffolds and subsequently underwent mock orthotopic surgery. Single-cell RNA sequencing of cells isolated from the tumor scaffolds highlighted two macrophage subpopulations characterized by increased gene expression of either C1qa, C1qb and Trem2 (Cq macrophages) or Chil3, Ly6c2 and Plac8 (Chil macrophages), respectively. Both populations were increased in the livers of tumor-bearing mice. Importantly, Chil3 macrophages were observed in the liver of tumor-bearing mice before overt metastasis occurred, suggesting pre-metastatic stromal reprogramming. Comparing the observed gene expression patterns to human PDAC samples, including primary tumor tissue, liver metastasis tissue and peripheral blood mononuclear cells (PBMCs) showed elevated Cq macrophage markers in those samples, indicating their potential as possible biomarkers; however, no elevation of Trem2 was detected in circulating PBMCs [65]. Additional studies are needed to better characterize how specific macrophage subpopulations are involved in tumor evolution and metastagenesis.

#### 2.2.2. Predominant Expansion of Specific Neutrophil Groups in Hepatic Metastasis

Neutrophils were previously regarded as a homogenous population that plays an integral part in the immune response and were therefore considered to be rather neutral in tumorigenesis and metastasis. A recent review discussed the role of neutrophils in pre-metastatic niche formation and ascribed neutrophils a leading role in that process while differentiating between anti- and pro-metastatic phenotypes, N1 and N2, respectively. It was suggested that the cytokine milieu around those neutrophils could determine their phenotype [66]. It is currently unclear whether an increased neutrophil count is a consequence of systemic inflammation during tumorigenesis or a contributor to pancreatic carcinogenesis and metastatic progression [67]. A recent study found that in KPC mice, liver metastases preferentially contained P2RX1^−^ neutrophils, which seemed to promote CD8^+^ T-cell exhaustion via the Nrf2/PD-L1/PD1 signaling axis [68]. Despite the lack of a clearly defined pre-metastatic window, this study supports the idea of the involvement of specific pro- and anti-tumoral neutrophils in metastasis formation.

Another study showed that TIMP-1 binding to CD63-expressing neutrophils and activation of ERK lead to formation of neutrophil extracellular traps (NETs) [15]. Further studies by the same group in KPC mice and human PDAC-derived tumor tissues showed a strong correlation between plasma TIMP-1 levels and NET burden in the TME. Moreover, the combination of TIMP-1 levels and NET burden was shown to have potential as a composite prognostic marker (TINE). The involvement of NETs in the metastatic process was previously pointed out in both liver-tropic colon cancer and lung-tropic breast cancer [69,70]. The exact mechanisms of neutrophil- and NET-mediated metastatic progression warrant further investigation.

### 2.3. Immunomodulation

Tumor progression is normally counteracted by intact immune responses. If the immune system fails to completely eradicate the tumor and restore homeostasis, evolution of the cancer cells though stochastic events and the process of natural selection leads to clones that evade anti-tumor immune responses, usually through a combination of processes that make them “invisible” to anti-tumor effector cells (e.g., CD8^+^ cytotoxic T lymphocytes (CTLs), NK cells, etc.) [71]. In a similar fashion, the metastatic process requires strategies to evade immunologic destruction of DTCs.

#### Tumor-Derived Immunosuppressive Factors Weaken the Anti-Metastatic Mechanisms of the Liver

A recent study showed that EVs derived from the human PDAC cell lines L3.6pl and TBO368 carry immunomodulatory factors, such as TGF-β1 [22]. In vitro testing showed uptake of those EVs by NK cells, which led to a significant decrease in their anti-tumorigenic function as determined by a decrease in activating receptors, decreased TNF-α and INF-γ production, impaired metabolism and decreased cytotoxicity against pancreatic cancer stem cells. TGF-β1, acting via induction of the SMAD2/3 signaling pathway, was shown to be a likely triggering factor of these observations. Further in vivo testing showed that those EVs were taken up by the liver of immunodeficient NSG mice. This suggests that tumor-derived EVs could cause a cascade, which ultimately leads to an impaired immune response by inhibiting NK cell function. A notable limitation of this study is the lack of in vivo experiments in immunocompetent mice; thus, the immunosuppressive effect of tumor-derived EVs on liver-resident NK cells in the context of the pre-metastatic niche awaits further investigation.

Mixed background B6129SF1/J mice orthotopically injected with the KPC-derived PDAC cell line LMP showed an increase in CD11b^+^ dendritic cells (DCs) in pre-metastatic and early-metastatic liver tissue compared to normal liver tissue [20]. Such CD11b ^+^ DCs expressed pro-tumoral mediators, including CCL2, CXCL1, CXCL2, IL-6 and TNF-α, as well as PD-L1 and PD-L2 surface proteins involved in immunosuppression, with the latter shown to inhibit CD8 T-cell responses. In vivo experiments identified tumor-derived GM-CSF as a factor involved in monocyte differentiation into CD11b^+^ DCs in the liver. These in turn increased the Treg:CD8^+^ T cell ratio by stimulation of Treg cells and concomitant PD-1 expression, which led to a suppressed CD8^+^ T-cell response. The above suggest an immunosuppressive role for CD11b^+^ DCs during metastagenesis. The study also revealed that CD11b^+^ DCs express both PD-L2 and MGL2, whereas either blockage of PD-L2 or depletion of MGL2^+^ CD11b^+^ DCs abolished their immunosuppressive and pro-metastatic effects. Similar DC markers were observed in human PDAC metastases.

Previous and recapitulated findings show that myeloid cells are one of the most abundant recruited cell populations to the tumor and (pre-)metastatic microenvironment. Conversely, cell populations of the adaptive immune response (CD8^+^ T cells and CD4^+^ T cells) decrease in numbers. Based on these findings, a recent study using rhabdomyosarcoma mouse models that metastasize to the lung employed deep transcriptional analysis to show an upregulation of pro-inflammatory genes (including MMP-9, S100A8 and S100A9) in the pre-metastatic lung that are associated with immunosuppression. Genes involved in functional T-cell responses were also downregulated. Genetically engineered myeloid cells were designed to secrete anti-tumorigenic IL-12 (IL12-GEMy). Treatment with IL12-GEMy increased CD8^+^ T cells, CD4^+^ T cells and NK cells, as well as associated INF-γ production and conventional DCs, which serve as activators of the adaptive immune response. Downregulated immunosuppressive pathways included TGF-β, IL-1, IL-6 and the IL-8-signaling pathway. In addition, MMP-9, CXCR4 and FN expression was decreased. IL12-GEMy significantly reduced metastasis and tumor progression in mice. In order to validate these observations for an epithelial tumor known to metastasize to the liver, the KPC PDAC mouse model was used. IL12-GEMy treatment delayed KPC primary tumor outgrowth and targeted the pre-metastatic niche of the liver [72]. In summary, the study implicates a myeloid-cell-heavy pre-metastatic niche microenvironment in suppression of adaptive immune responses and provides a therapeutic strategy using IL12-GEMy, which can reverse this suppression by reinvigorating anti-metastatic CD8^+^ T-cell responses.

Circulating tumor cells (CTCs) in the portal circulation strongly correlate with the occurrence of hepatic metastases [73]. In vivo and ex vivo, CTCs were observed to cluster with myeloid-derived fibroblasts (M-Fb) in the portal circulation of PDAC patients. Compared to isolated CTCs, CTC/M-Fb co-cultures enhanced CTC proliferation and motility. Further testing showed that anti-colony stimulating factor receptor 1 (anti-CSFR1), anti-IL-8 and employment of anti-IL-34 significantly surpressed myeloid cell to MDSC/M-Fb differentiation markedly inhibited CTC/M-Fb cluster formation and increased CTC apoptosis. The anergic state of portal blood T cells was reversed by anti-CSFR1, anti-IL8 and anti-IL-34 [74]. Further research is needed to assess whether curbing CTC expansion and CTC/M-Fb interaction in portal circulation modulates the risk of establishing pre-metastatic niches and hepatic metastatases.

### 2.4. Organotropism

Pre-disposed metastatic spread to specific organs is directly related to pre-metastatic niche formation. The underlying molecular pathways causing this bias have been the focus of investigation in recent years. In the case of pancreatic cancer, it has long been postulated that organotropism is related to anatomic reasons relating to portal circulation draining the venous outflow from the pancreas directly into the liver. However, a recent study found that TDSFs also contribute to this process [23]. Specifically, hepatic predilection was shown to be heavily influenced by integrin expression patterns on tumor-derived exosomes. Liver-tropic BxPC-3 cells secreted exosomes enriched in integrin α5β5 (ITGα5β5), which fused mainly with hepatic Kupffer cells. This led to upregulation of the pro-metastatic S100A8 and S100P genes in Kupffer cells and was associated with an FN-rich liver microenvironment—findings that are consistent with hepatic pre-metastatic niche development, as was previously demonstrated by the same group [16].

Another study showed that the protein p120ctn, expressed by PDAC cells and known to play a role in intercellular adhesion and stabilization of E-cadherin, influences metastatic organotropism in PDAC. Biallelic loss of p120ctn led to lung metastasis, whereas cells with monoallelic p120ctn formed liver metastasis, highlighting the fact that not only the quality of pro-metastatic factors matters but also potentially the quantity [75].

The metabolic milieu of different organs may be another determinant of organotropism. By comparing autochthonous KPC tumors to subcutaneously transplanted KPC tumors, Sullivan et al. demonstrated significant differences in the metabolic composition of the tumor interstitial fluid [76]. Furthermore, a recent report identified CDKN2A/CDKN2B co-deletion in human PDAC (resulting in loss of p16/p15) as a factor predisposing to liver metastasis through the induction of metabolic alterations that enable growth within the liver milieu [77]. Knockdown of p16/p15 in PDAC cells led to upregulation of several genes involved in ammonia consumption (including glutamine synthetase, GLUL), as well as concurrent downregulation of genes associated with deamination, including glutamine deaminase (GLS2). As a consequence, PDAC cells became less sensitive to glutamine depletion and high ammonia levels—the conditions encountered in the hepatic milieu.

### 2.5. Angiogenesis and Vascular Permeability

Successful growth of tumors and metastasis is highly dependent on effective neo-angiogenesis, as well as non-angiogenic mechanisms, such as vessel co-option and vasculogenic mimicry, in order to overcome the hypoxic conditions in PDAC [78]. Different angiogenic factors, such as VEGF, IL-8, PD-ECGF and HGF, have been implicated in the growth of liver metastases [79]. Various myeloid cells, including neutrophils, dendritic cells, monocytes and macrophages, may also contribute to tumor angiogenesis through production of proangiogenic factors or inhibition of antiangiogenic factors [80]. Increased vascular permeability aids in tumor propagation by enabling tumor extravasation and intravasation [81].

In the past, circular RNAs (circRNAs) were considered a byproduct of the mRNA transcription process with no functionality. More recent research ascribes functionality to these circRNAs, for example, as potentiators or inhibitors of certain microRNAs (miRNAs), whereby those miRNAs themselves have been linked to a variety of cancers [82,83,84]. Various circRNAs differ in terms of their effect on cancers. In PDAC, they can drive tumor growth and metastasis [85]. It was found that exosomes derived from Hs766 T-(L2) and AsPC-1 human PDAC cell lines contain circ-IARS. Subsequently, in vitro observations showed that circ-IARS absorbs miR-122 after being taken up by human umbilical vein endothelial cells (HUVECs). In a modified transwell assay, an endothelial monolayer with HUVECs was generated, and cancer cells were placed on top to assess transendothelial migration. Co-administration of exosomes promoted increased permeability of the HUVEC monolayer mediated by RhoA and F-actin upregulation and ZO-1 downregulation, leading to increased migration of the seeded cancer cells. In vivo experiments showed decreased liver metastasis and tumor size in circ-IARS knockdown mice [86]. Therefore, it appears that PDAC-derived exosomes carrying circ-IARS can promote vascular permeability in the liver, which may contribute to pre-metastatic niche and metastasis development. Further research could demonstrate the exact temporospatial occurrence of this observation.

In vitro studies showed that activated HSC found in the pre-metastatic niche can lead to increased angiogenesis when co-cultured with T3M4 human PDAC cells. This effect is mediated by the expression of CXCL8 (IL-8) and CCL2 (MCP-1), which have a dual pro-inflammatory and pro-angiogenic function [87]. The evidence outlined above suggests a mechanism whereby PDAC cells signal secondary organ MAMs, setting in motion pro-angiogenic and ultimately pro-metastatic effects.

## 3. Clinical Translation: Diagnostic and Therapeutic Opportunities

Pre-metastatic niche formation and its clinical impact have been met with increasing interest in recent years. The process of pre-metastatic niche preparation may provide a window of diagnostic and therapeutic opportunity. Detection of changes that favor metastatic colonization in uninvolved organs can potentially stratify patients as high risk for future metastasis at those sites, whereas phenotypic alterations that may counteract metastases (e.g., signs of potent anti-tumor immunity) may predict low probability for metastases. Moreover, therapeutic interventions targeting core pathways involved in pre-metastatic niche development and reprogramming of immune elements from pro-metasttic to anti-metastatic may aid in metastasis prevention.

### 3.1. Detection by Molecular Imaging and/or Biopsy

The approaches to detect the pre-metastatic niche can be classified as either “niche-agnostic” or “niche-informed”. Niche-agnostic approaches essentially attempt to quantify alterations in seemingly uninvolved organs and provide a metastasis score without focusing on specific molecules, cells or biologic processes that are deregulated during pre-metastatic generation. Radiomics—or quantitative image analysis—is evolving as one such approach [88]. It involves extraction of data from cross-sectional imaging studies (e.g., contrast-enhanced CT or MRI) and calculation of variables that are derived from the comparison of individual pixels to their surroundings. These lead to generation of quantitative imaging features (QIFs) that act as biomarkers to represent the digital “texture” of the organ and may relate to underlying biologic processes, such as angiogenesis, inflammation, ECM remodeling, etc. Combination of QIFs in prediction models, especially when generated with machine learning methods, can yield robust data. Within this framework, our ongoing work suggests that quantitative image analysis of preoperative CT scans of patients with resectable PDAC can predict early metastases [89].

Niche-informed approaches attempt to detect specific features that are associated with pre-metastatic niche formation (or its absence). Certain molecules that are upregulated within the pre-metastatic niche may be detectable using imaging. For example, VLA4 (ITGα4β1) and S100A8/A9 have been employed for radiologic detection of the pre-metastatic niche [90,91]. VLA4-positive BMDCs were visualized with PET using ^64^Cu-CB-TE2A-LLP2A, and SPECT was employed using S100A8/9-specific ^111^In-labeled antibodies. Zhang et al. demonstrated successful PET imaging in a mouse model of PDAC using CCL2-tagged nanoparticles radiolabeled with ^64^Cu and loaded with gemcitabine [92]. These nanoparticles targeted CCR2, which is involved in the recruitment of neutrophils and inflammatory monocytes in tumors and metastases, thus making it a potential candidate for pre-metastatic niche imaging. Such an approach can theoretically be expanded to other chemokine receptors with crucial roles in pre-metastatic niche formation, such as CXCR2.

Along similar lines, Farahi et al. demonstrated that ^111^In-tropolonate can be used to radiolabel patient-derived neutrophils and track them while they home into lung tumors using SPECT-CT [93]. Given the involvement of neutrophils in pre-metastatic niche formation, such an approach may be applicable for its detection. Conversely, tracing effector lymphocytes such as T cells and NK cells may be suitable for detection of potent “antimetastatic” niches that are likely to counteract metastagenesis (although this speculation remains to be tested experimentally). This can be accomplished using either radiolabeled patient-derived lymphocytes or by infusing radiolabeled antibodies or other molecules binding such cells [94].

Lastly, the existence of pre-metastatic niches may be predictable by quantification of some of its mediators, including molecules such as TIMP-1, as well as EVs. As mentioned above, PDAC cell-derived GFP-labelled exosomes have been shown to disseminate to the liver, lungs and bones. Similarly, a study involving breast cancer cell-derived exosomes using an orthotopic nude mouse model showed that GFP-labelled exosomes disseminated to tumor-associated cells at metastatic sites [95]. Another study showed that in vitro radio-labelled MDSCs, upon adoptive transfer into melanoma or breast cancer mouse models, accumulated at primary and metastatic tumor sites [96]. Moreover, EVs have gained popularity as analytes for liquid biopsy for different human cancer types [97,98]. Although their isolation can be complex, the wealth of information contained therein may allow for risk stratification and prediction of organotropism.

It has been suggested that DTCs, which may go undetected in common laboratory settings, are possible contributors to the niche-forming process [99]. Furthermore, the presence of increased TIMP-1 in pre-malignant lesions, such as chronic pancreatitis, raises the question as to how early pre-metastatic niche formation starts. Previous studies showed that circulating pancreatic cells were detectable in the bloodstream and livers of mice with pancreatic intraepithelial neoplasia (PanIN) and in the bloodstream of humans with pancreatic cystic lesions prior to clinical tumor manifestation [100,101]. Further studies elucidating mechanisms by which DTCs influence their microenvironment, evade immunosurveillance and eventually develop into overt metastases will open up possible theranostic opportunities. Moreover, studies investigating the occurrence of possible hepatic pre-metastatic niches in pre-malignant pancreatic conditions such as PanIN, intraductal mucinous cystic neoplasms or chronic pancreatitis could contribute to the understanding of the metastatic cascade in PDAC and present an opportunity for early diagnosis of potential future metastatic sites.

### 3.2. Therapeutic Aspects of the Pre-Metastatic Niche

Therapeutic interventions aimed at disrupting the pre-metastatic niche can theoretically mitigate the risk of metastasis by making the “soil” no longer “fertile”. Moreover, they may play a role in counteracting resistance to anti-cancer therapies, as disseminating cancer cells may find shelter in such established niches, assume a dormant state and emerge later as metastases either in the same or in tertiary organs [102].

**Figure 2:**
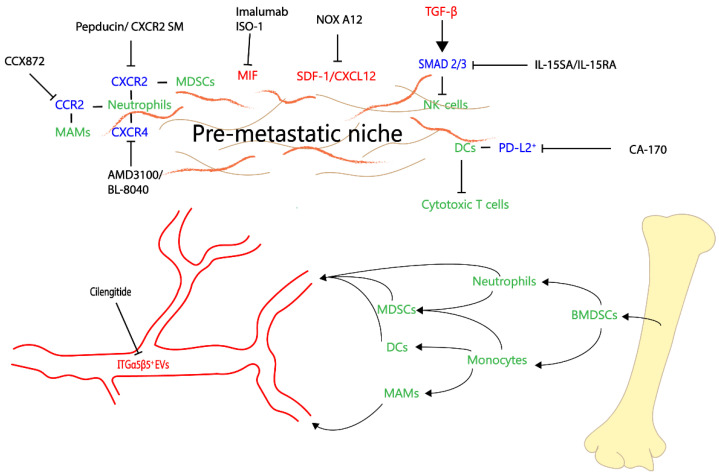
Potential therapeutic targets of the hepatic pre-metastatic niche. Integrin α5β5-expressing tumor-derived EVs inhibited by Cilengitide. CXCR4 inhibitors plerixafor or BL80-40 can inhibit CXCR4^+^ neutrophils. CCX872 inhibits CCR2^+^ MAMs and neutrophils. Pepducin or CXCR2 small-molecule inhibitor (AZ13381758) blocks CXCR2^+^ neutrophils and MDSCs. MIF inhibitors imalumab and ISO-1. SDF-1/CXCL12 inhibitor NOX A12. IL-15 superagonist (SA)/IL-15 receptor alpha complex rescues the SMAD2/3-mediated inhibitory effects of TGF-β on NK cells. PD-L1/2 inhibitor CA-170 inhibits PD-L2-mediated inhibition of CD8^+^ T cells.

Conceptually, the pre-metastatic niche may be targeted therapeutically via inhibition of TDSFs in an attempt to abrogate its formation and by manipulation of immunomodulatory, inflammatory, metabolic and stromal changes in order to prevent or reverse pre-metastatic niche generation. This is supported by several studies reviewed above, which showed that genetic or pharmacologic inhibition of certain cellular or molecular pre-metastatic niche promoters, such as neutrophils, TIMP1, MIF or IL-6, halted pre-metastatic niche initiation or halted its progression [12,16] (Figure 2).

Anti-IL-6 monoclonal antibody monotherapy with siltuximab was tested in a basket trial that included metastatic pancreatic cancer patients but failed to improve patient outcomes [103] (Table 2). This demonstrates, among other things, that timing of delivery of such targeted agents may be critical and that multi-agent therapy may be necessary. A phase II clinical trial involving a combination of gemcitabine/nab-Paclitaxel chemotherapy with the anti-IL-6R monoclonal antibody tocilizumab in patients with locally advanced or metastatic PDAC is ongoing [104]. A recent phase I trial demonstrated the safety of imalumab (BAX69), a monoclonal antibody inhibitor of oxidized MIF, in colorectal, ovarian and lung cancer [105]. Another MIF inhibitor, ISO-1, was shown to inhibit pancreatic cancer cell invasion, migration and proliferation in vitro, as well as tumor growth in vivo in an immunodeficient murine model [106]. Given the importance of MIF in PDAC pathogenesis and pre-metastatic niche formation, inhibiting its effects on pre-metastatic niche formation in the neoadjuvant or perioperative setting may prove efficacious in reducing metastases [107].

CCR2 inhibition was previously shown to counteract metastasis and tumor growth in a murine model [64]. The CCR2 inhibitor CCX872 was administered to patients with locally advanced or metastatic PDAC in combination with FOLFIRINOX chemotherapy and proved to have a good safety profile, as well as increased overall survival, compared to FOLFIRINOX alone [108]. 

Previous research indicated an involvement of the SDF-1/CXCL12–CXCR4 axis in tumorigenesis and metastatic progression by enhancing tumor cell migration towards the liver along a CXCL12 gradient, as well as by inhibiting tumor cell and CD8^+^ T-cell interaction in the TME [112,113]. Based on these insights, clinical trials in human PDAC have tested both inhibitors of SDF-1/CXCL12 and CXCR4 in combination with a PD-1 inhibitor, with encouraging results [109,110,114]. It is noteworthy that MIF can also ligate CXCR4; therefore, CXCR4 inhibitors may be efficient against MIF-mediated pathways. On the contrary, this redundancy of multiple parallel pathways may explain why monotherapy with targeted agents is often unsuccessful, emphasizing the need for combination therapies to prevent escape through parallel pathways. Attesting to that redundancy, a recent study found that in PDAC, inhibition of TAM recruitment via CCR2 blockade leads to a compensatory increase in CXCR2^+^ neutrophils and vice-versa [115]. However, combined CCR2 and CXCR2 blockade led to a sustained inhibition of systemic mobilization of both CCR2^+^ TAMs and CXCR2^+^ neutrophils, which enabled anti-tumor immune response and potentiated FOLFIRINOX chemotherapy. This strategy may be extrapolated to the recruitment of the above myeloid cell subsets to pre-metastatic niches and warrants further research.

Immunomodulatory effects on NK cells induced by exosome-derived TGF-β1 were neutralized by the use of IL-15SA/IL-15RA fusion complex, which restored NK cell functionality in vitro in human cancer cell lines [116]. This presents a potential opportunity for therapeutic intervention, although the potential tumor-suppressive effects of TGF-β during early carcinogenesis should be considered [117]. We previously mentioned that an immunosuppressive DC subset was responsible for impaired CD8^+^ T-cell response, which could be rescued by inhibition of PD-L2 [20]. A phase I clinical trial is ongoing investigating the effects of the PD-L1/2 inhibitor CA-170 in patients with advanced solid tumors [118].

Counteracting EV-mediated pre-metastatic niche formation may be feasible either through inhibition of their production or through blockade with EV-targeting agents that would promote their elimination. Inhibiting Rab27 GTPases involved in exosome secretion is an attractive strategy [21,119]. A new EU-sponsored CORDIS project (project ID 890900) will investigate Rab27a as a novel anti-cancer target. Integrin α5β5 is involved in PDAC cell-derived exosome homing to the liver, where it leads to establishment of a pre-metastatic niche [23]. Cilengitide is a selective inhibitor of integrins α5β3 and α5β5 and has previously been used in combination with gemcitabine in patients with unresectable PDAC, although it did not improve the overall outcome [111]. The use of cilengitide in earlier stages of pancreatic cancer, for instance in the perioperative setting, could potentially attenuate the risk of future metastases.

An interesting example as to how nanoparticles or exosomes could be used as drug-carrying vehicles in a therapeutic attempt to target pre-metastatic niche formation was shown in a study with a murine model of spontaneous breast cancer [120]. Melittin, a cationic host defense peptide involved in immune responses, occurs naturally in the honeybee. A combination of a peptide nanoparticle and melittin (α-melittin-NP) was shown to activate LSECs and was able to suppress metastatic outgrowth in the liver and lungs in a spontaneous breast cancer mouse model. Specifically, α-melittin-NP modulation of LSECs led to the recruitment of NK and T cells, resulting in anti-tumorigenic effects. Chemokine and cytokine expression of activated LSECs demonstrated upregulation of known pro-tumoral molecules, such as IL-1, CXCL9/10 and 13, among others. Therefore, LSECs represent potential therapeutic targets, as they are involved in various aspects of the metastatic process, including inflammation, immunomodulation and angiogenesis.

## 4. Conclusions: Obstacles and Future Directions

Remarkable progress has been made over the last decade with respect to elucidating the mechanisms of pre-metastatic niche generation. This has generated a multitude of opportunities for diagnosis and treatment, as well as many questions. Research on the hepatic pre-metastatic niche in PDAC appears to have focused primarily on stromal reprogramming and inflammation, whereas some emerging areas, such as immunometabolism and vascular remodeling, are understudied and deserve more attention. Furthermore, the majority of studies have been performed in murine models, and human data are scant. Murine models allow for near-complete manipulation of the experimental conditions, yet they do not fully recapitulate the biologic diversity observed in human PDAC, with some tumors being more aggressive than others. Additionally, lung tropism is overrepresented in many murine metastasis models, including the PDAC KPC model. Moreover, the immune system of mice has important differences compared to humans: immune cells bare different phenotypic markers, making direct translation impossible in certain cases (e.g., profiling of myeloid cells), whereas chemokines and other TDSFs may differ fundamentally, both in their receptor targeting and their function. Although several studies have made use of human PDAC cells, either in the form of cell lines or directly derived from patient material, their use in xenograft models is associated with the fundamental disadvantage of a lack of a fully functional immune system, as recipient mice must be immunocompromised for the cancer cell not to be rejected. Combining animal models and human material when possible is probably the most prudent approach in order to mitigate some of the aforementioned caveats. Lastly, comparison of findings among different cancer types with similar behavior (e.g., gastrointestinal cancers that metastasize to the liver) may contribute to a broader understanding of the metastatic process and allow for therapeutic targeting. Further investigation of the pre-metastatic niche is imperative, and we anticipate that it will lead to many exciting discoveries.

## Figures and Tables

**Table 1:** Pre-metastatic-niche-promoting factors and described modes of action.

Factor	Source	Target	Mode of Action	Experimental Model	Ref.
**VEGF**	Cancer cells	VEGFR1^+^ HPCs	Promotes homing of VEGFR1^+^ HPCs to the lung, leading to pre-metastatic niche formation	C57BL/6 mice with intradermal LLC and B16 cell injection	[11]
**PLGF**	Cancer cells	VEGFR^+^ HPCs	Promotes homing of VEGFR1^+^ HPCs to the lung, leading to pre-metastatic niche formation; redirects metastasis	C57BL/6 mice with intradermal LLC and B16 cell injection	[11]
**SAA**	Hepatocytes	Neutrophils; HSCs	Increases hepatic parenchymal FN and collagen I production, as well as Ly6G^+^ myeloid cell infiltration into the pre-metastatic niche	C57BL/6 SAA^−/−^ mice with orthotopic PDAC injection	[12]
**TIMP-1**	Cancer cells	HSCs; neutrophils	Activates HSCs by signaling via the CD63 receptor, causing release of SDF-1, leading to neutrophil accumulation in the pre-metastatic niche; binds CD63 on neutrophils to promote ERK-mediated NET formation	C57BL/6 mice with intravenous PC 9801L cell injection + AdTIMP-1 transduction; KPCxC57BL/6 TIMP-1^−^ mice + AdTIMP-1 transduction	[13,14,15]
**Tumor-Derived Exosomes Containing MIF**	Cancer cells	F4/80^+^ macrophages (KCs); Gr-1^+^ neutrophils	Uptake by KCs stimulates TGF-β release, which in turn induces FN production by activated HSCs and recruitment of additional BMDCs (macrophages and neutrophils) in the pre-metastatic niche	Intravenous injection of exosomes from PAN02 or KPC cell lines (exosome “education”) to C57BL/6 mice	[16]
**Tumor-Derived Exosomes**	Cancer cells	F4/80^+^ macrophages; CD11b^+^Gr-1^+^ MDSCs;	PAN02-H7 exosomes promote immune cell recruitment and upregulation of FN, S100A8 and S100A9 in the pre-metastatic liver; potentially CXCR4- and MMP-9-mediated	Intravenous injection of exosomes from PAN02 or PAN02-H7 (highly-metastatic) cell lines to C57BL/6 mice	[17]
**CXCR2 Ligands**	Peri-tumoral microenvironment	F4/80^+^ macrophages; NIMP1^+^ neutrophils; S100A9^+^ MDSCs	Promotes recruitment of MDSC and neutrophils in the hepatic pre-metastatic niche	KPC mouse model	[18]
**Granulin**	Bone-marrow-derived inflammatory monocytes	HSCs	Activates HSCs; activated HSCs release ECM proteins, such as periostin, thus inducing liver fibrosis and supporting metastasis	C57BL/6 mice with intrasplenic KPC/PAN02 cell injection	[19]
**GM-CSF**	Cancer cells	CD11b^+^ DCs	Stimulates BMDC to CD11b^+^ DC differentiation in the liver, which in turn directly inhibits CD8^+^ T cells via PD-L2 and indirectly via induction of Treg proliferation	B6129SF1/J mice with intrapancreatic LMP cell injection	[20]
**Rab27a**	Cancer cells	Recruited myeloid cells	Required for exosome production; aids in myeloid cell expansion both within the primary tumor and within the hepatic pre-metastatic niche	C57BL/6 mice with orthotopic/intrasplenic KPC cell injection	[21]
**TGF-β1 Within Exosomes**	Cancer cells	NK cells	Impairs NK cell functionality by downregulation of activating receptors, decrease in NK cell cytokine release and metabolic debilitation	In vitro testing of NSG mice	[22]
**ITGα5β5 on Exosomes**	Cancer cells	Kupffer cells	Activates Src and upregulates S100A8 and S100P expression	C57BL/6 mice with intravenous injection of human BxPC-3-derived exosomes	[23]

**Table 2:** Summary of clinical trials involving agents with pre-metastatic niche-modulating properties.

Agent	Combination Agent(s)	Target	Cancer Type(s)	Trial ID	Study Type	N	Endpoints/Outcomes	Remarks	Ref.
Siltuximab	-	IL-6	mPDAC CRC NSCLC H&N	NCT 00841191	Phase I/II basket trial	84	0% ORR 6% SD	15% hepatic function abnormalities	[103]
Tocilizumab	Gemcitabine, Nab-Paclitaxel	IL-6R	laPDAC mPDAC	NCT 02767557	Phase II	147	OS	ongoing	[104]
CCX872-B	FOLFIRINOX	CCR2	laPDAC mPDAC	NCT 02345408	Phase I	50	29% OS at 18 months	better OS associated with lower peripheral blood monocyte counts results not final	[108]
Olaptesed/NOX-A12	Pembrolizumab	CXCL12	mPDAC mCRC	NCT 03168139	Phase I/II	20	25% SD 1.9 months median PFS 42% OS at 6 months 19% OS at 12 months	median T-cell density at invasive margin 327 cells/mm^2^ results not final	[109]
Motixafortide/BL-8040	Pembrolizumab (Cohort 1) + Irinotecan, 5FU + LV (Cohort 2)	CXCR4	mPDAC	NCT 02826486	Phase II	80	Cohort 1, 3.4% ORR Cohort 2, 32% ORR	ongoing, results not final	[110]
Cilengitide	Gemcitabine	Integrin α5β3 and α5β5	laPDAC mPDAC		Phase II	86	6.7 months mOS 3.6 months PFS 17% ORR		[111]

CRC, colorectal cancer; H&N, head and neck cancer; NSCLC, non-small cell lung cancer; laPDAC, locally-advanced PDAC; mPDAC, metastatic PDAC; ORR, overall response rate; OS, overall survival; PFS, progression-free survival; SD, stable disease.

## Abbreviations

BMDC	bone-marrow-derived cells
CAF	cancer-associated fibroblasts
DC	dendritic cells
DTC	disseminated tumor cells
CTL	cytotoxic T lymphocytes
CTC	circulating tumor cells
ECM	extracellular matrix
EVs	extracellular vesicles
FN	fibronectin
HPCs	hematopoietic progenitor cells
HSCs	hepatic stellate cells
HUVECs	human umbilical vein endothelial cells
LSECs	liver sinusoidal endothelial cells
KCs	Kupffer cells
KPC	LSL-Kras^G12D/+^;LSL-Trp53^R172H/+^;Pdx-1-Cre genetically engineered mouse model of PDAC with the genotype ([46])
M-Fbs	myeloid-derived fibroblasts
MDSCs	myeloid derived suppressor cells
MIF	macrophage migration inhibitory factor
MMP	matrix metalloproteinase
NKs	natural killer cells
PDAC	pancreatic ductal adenocarcinoma
PDECs	pancreatic ductal epithelial cells
PLGF	placental growth factor
PSCs	pancreatic stellate cells
SAA	serum amyloid A
SDF-1	stromal cell-derived factor 1 (also known as CXCL12)
TAMs	tumor-associated macrophages
TDSFs	tumor-derived secreted factors
TGF-β	transforming growth factor-β
TIMP-1	tissue inhibitor of metalloproteinases,
TME	tumor microenvironment
Treg	regulatory T cells
VEGF	vascular endothelial growth factor
